# Review on the impacts of indoor vector control on domiciliary pests: good intentions challenged by harsh realities

**DOI:** 10.1098/rspb.2024.0609

**Published:** 2024-07-24

**Authors:** Christopher C. Hayes, Coby Schal

**Affiliations:** ^1^ Department of Entomology and Plant Pathology, North Carolina State University, Campus Box 7613, Raleigh, NC 27695-7613, USA

**Keywords:** malaria, vector control, malaria elimination, indoor pests, bed bugs, cockroaches

## Abstract

Arthropod vectored diseases have been a major impediment to societal advancements globally. Strategies to mitigate transmission of these diseases include preventative care (e.g. vaccination), primary treatment and most notably, the suppression of vectors in both indoor and outdoor spaces. The outcomes of indoor vector control (IVC) strategies, such as long-lasting insecticide-treated nets (LLINs) and indoor residual sprays (IRSs), are heavily influenced by individual and community-level perceptions and acceptance. These perceptions, and therefore product acceptance, are largely influenced by the successful suppression of non-target nuisance pests such as bed bugs and cockroaches. Adoption and consistent use of LLINs and IRS is responsible for immense reductions in the prevalence and incidence of malaria. However, recent observations suggest that failed control of indoor pests, leading to product distrust and abandonment, may threaten vector control programme success and further derail already slowed progress towards malaria elimination. We review the evidence of the relationship between IVC and nuisance pests and discuss the dearth of research on this relationship. We make the case that the ancillary control of indoor nuisance and public health pests needs to be considered in the development and implementation of new technologies for malaria elimination.

## 1. Introduction

### (a) The global burden of vector-borne diseases

Vector-borne diseases (VBDs) have directly impacted global populations through high morbidity and mortality, their use as weapons, and more fundamentally, by impeding human development and innovation [1]. Examples of these impacts range from the introduction of malaria to susceptible populations during colonization to increasing reports of VBD coinfections [2–4]. Currently, more than four out of every five people are at risk for VBD transmission, resulting in over 1 billion cases of VBDs annually, that account for nearly one-sixth of all global illness and disability [5].

**Figure 1.**
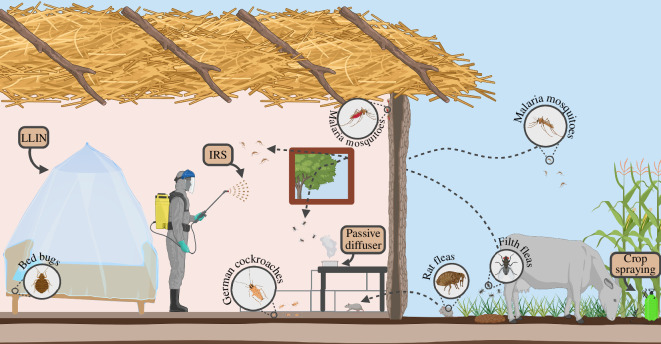
The overlap of IVC tactics and domiciliary pests. The success of IVC in malaria-endemic communities can be impacted by obligate domiciliary pests and transient pests that occasionally enter the home. Outside the home, highly mobile insects (house fly, *Musca domestica*; flea, *Ctenocephalides* and *Xenopsylla* spp.) are exposed to insecticides outdoors via targeted or crop sprays. In the home, obligate indoor pests with low mobility (bed bug, *Cimex* spp.; German cockroach, *Blattella germanica*) and those that have entered the home from outside are exposed to insecticides from consumer products (e.g. passive insecticide diffusers, aerosols), IRS and ITNs, including LLINs. Each of these non-target exposures exerts strong selection pressure on indoor pests in malaria endemic communities, leading to the widespread emergence of insecticide-resistant pest populations, which greatly hinder and disrupt vector control programmes. Figure created with BioRender.com. LLINs, long-lasting insecticide treated nets; IRS, indoor residual spray; ITN, insecticide treated bed net.

The overwhelming burden of VBDs has spurred the advent of control measures following two broad strategies: (i) treatment of known and potential disease hosts to eliminate disease reservoirs and (ii) control of arthropod vectors [6–8]. While these are interdependent strategies, vector control has played a greater role in the global mitigation of VBDs, as it has been the principal (and sometimes only) method of disease management available [9]. Vector control can be divided into two sub-categories, namely outdoor and indoor vector control (IVC). Outdoor or exterior vector control practices focus heavily on vector habitat and breeding site management and disruption, community education and behaviour modification, larvicide and adulticide applications and oviposition site monitoring [10–12]. For the past several decades, IVC has relied on the distribution, use and application of insecticide-based products within homes, primarily targeting *Anopheles* mosquitoes, the vectors of malaria (figure 1) [7].

### (b) Control of vectors indoors

Historically, IVC measures have included interior applications of insecticides including dichlorodiphenyltrichloroethane (DDT) and more recently pyrethroids, as well as the widespread distribution of insecticide-treated nets (ITNs) and long-lasting insecticide-treated nets (LLINs). These measures have been primarily aimed at disrupting the transmission of malaria, which persists as the most significant VBD globally [13]. The two bed net types differ in their preparation, with ITNs (historic) being treated by dipping with any World Health Organization (WHO)-recommended insecticide post-manufacturing and requiring frequent retreatment, while LLINs (modern) are manufactured using WHO-recommended insecticide impregnated fibres designed to last up to 3 years without reimpregnation, making LLINs the most widely adopted indoor intervention today [14]. The use of LLINs and indoor residual sprays (IRSs) are integral components of current and historic malaria vector control programmes (figure 1), with the majority of their deployment occurring throughout Africa, where together they are responsible for over 80% of mitigated malaria cases in Africa from 2000 to 2015 [15–17].

Historically, all WHO-recommended ITNs have been treated exclusively with pyrethroid insecticides (IRAC class 3) [18], modelled after the naturally occurring pyrethrins that target the voltage-gated sodium channel in the insect nervous system and considered relatively safe for human exposure [19]. Pyrethroids, and other newly used active ingredients (AIs), including those such as broflanilide, which represent novel modes action aimed at combating resistant vector populations, have replaced DDT and other organochlorine insecticides in IRS applications [20,21]. Similarly, recent research and funding for LLIN development have gone beyond pyrethroid-only formulations and are focused on combinations of AIs and synergists, such as co-impregnation with pyrethroids and chlorfenapyr (Interceptor® G2 and PermaNet Dual), pyriproxyfen (Royal Guard) and piperonyl butoxide (PBO) (PermaNet 3.0) [22–25]. Despite these recent advances in net design, of the nearly 2 billion distributed treated nets in the past decade, the vast majority are single ingredient pyrethroid-impregnated formulations [26]. Therefore, while efforts to replace nets with dual-ingredient and synergist chemistry are well underway, product development, lag time, supply chain issues and increased user cost will necessitate the continued reliance on single-ingredient pyrethroid-treated nets for the near future.

### (c) Interdependency of vector and pest control

Although the continued programmatic motivation in the development, distribution, application and evaluation of IVC tools is the reduction of malaria incidence and burden, this is not always the case from the perspective of the user communities. It has been shown for decades that while communities appreciate the long-term health benefits associated with decreased malaria burden, they place as high or higher value on the practical, immediate and tangible relief from indoor nuisance and biting pests [27]. Indeed, this predilection has been shown by several studies to promote the uptake and adherence to IVC practices by user communities; conversely, when the ancillary benefit of pest control is lost, community perception and adherence to IVC procedures diminishes [28–39]. Yet, a dearth of modern research has investigated the impacts of IVC on indoor pest populations, and subsequently, how domiciliary pest control affects IVC. Most importantly, the risks of abandonment of IVC tactics owing to failure to secondarily control domiciliary pests has received little attention. Therefore, we have highlighted the current scarcity of research on this topic and present a case for considering pest management during the design and implementation of the next generation of IVC tools.

### (d) Document identification

**Figure 2.**
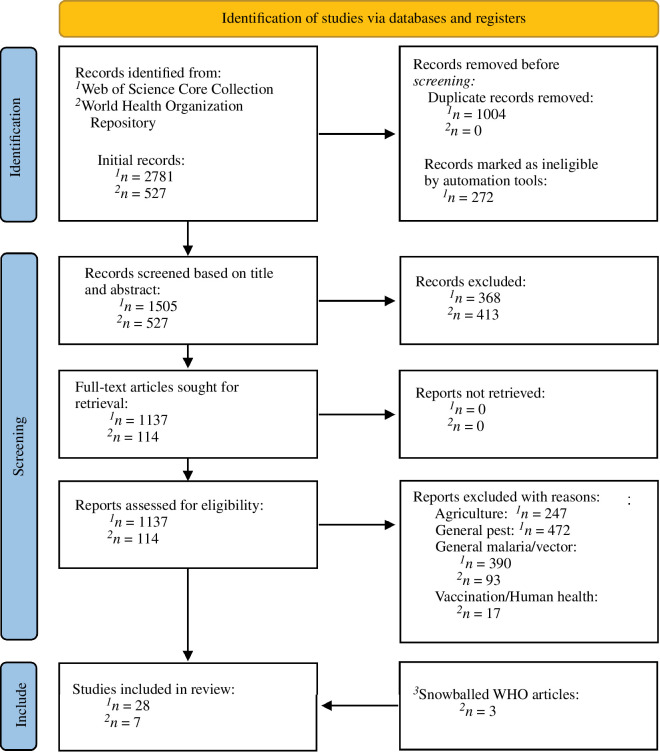
Flowchart of the selection process for articles related to the interaction of domiciliary pests with IVC tools. Comprehensive searches were performed following the PRISMA guidelines as closely as possible (see http://www.prisma-statement.org/) [40] across two repositories: Web of Science Core Collection and the World Health Organization Publication Repository. Searches covered publications spanning from 1900 to August 2023. ***^1^*** In reference to records obtained from the Web of Science Core Collection Database. ***^2^*** In reference to records obtained from the World Health Organization Publication Repository. ***^3^*** Snowballing: ‘Reviewing the citations of systematically identified relevant publications for additional material.’ [41].

To identify studies, we conducted a literature review using the Web of Science (WoS) Core Collection database as well as the WHO Repository for publications relating to both IVC programmes and domiciliary pests (figure 2). We searched the relevant literature from 1900 to current publications (29 August 2023) using the following search terms. For the WoS Core Collection: Search 1: ALL FIELDS: (malaria) AND ((bed bug OR bedbug*) OR (cockroach*) OR (filth flies) OR (flea*) OR (nuisance) OR (pest)) OR ((bed bug OR bedbug*) AND (net*)) resulting in 1250 records; and Search 2: TITLE only: (malaria) AND ((indoor residual spray) OR (bed*) OR (net*)) OR (malaria) AND (bednet) OR (bednet*) resulting in 1531 records. For the WHO Repository (1990–current): HEALTH TOPIC = ‘Malaria’ OR ‘Vector Control’ OR ‘Vector-borne diseases’ OR ‘Pesticides’ OR ‘Insecticide treatments’ resulting in 527 records. Studies and publications were included if they related to the explicit interaction of domiciliary pests with IVC tools, contained quantified or anecdotal accounts of these interactions, or if they discussed the societal or programmatic implications of these interactions. Non-automated record screening was performed by C.C.H., with final article selection confirmed by C.S., with disagreement on relevancy and inclusion discussed until agreement was reached. While a historical bias may exist in regions covered by peer-reviewed material, potentially limiting the scope of available relevant publications, the authors searched without the use of language or journal filters in an attempt to ensure that no relevant articles were excluded from this review.

Initial screening was performed to remove duplicate records, with minimal reliance on automated tools for screening (figure 2). Following initial screening, further assessment of search results was first done based on article topic, followed by title and abstract, with non-applicable records being dropped. This led to a total of 1248 records, which were retrieved, and the full text screened for relevance. Full-text screened publications were excluded if they related strictly to agricultural or beneficial non-target insects, general pest management and biology, general malaria or vector control or vaccination and human health. This process removed a total of 1219 records. Additionally, we identified three relevant WHO publications not present in the WHO repository through snowballing of identified publications [41]. We identified a final total of only 28 peer-reviewed publications that met our search criteria, spanning from 1971 to 2022 [28–32,34–39,42–58], and an additional seven WHO publications spanning from 1968 to 2022 [3,5,26,59–62]. It is important to note that these expansive criteria likely fail to capture historic publications from researchers in frequently underrepresented regions and countries which may be excluded from modern research repositories.

## 2. An intertwined history: suppression of domiciliary pests fosters indoor vector control adoption

To understand the association between domiciliary pest control and the success of IVC programmes, it is important to discuss their long and codependent history. One of the earliest programme evaluations, a blinded survey of nuisance arthropods in Gambian homes, performed in the late 1980s, surveyed 361 homes spanning six villages for bed bugs, ticks and the presence of headlice on children. All participating homes were provided untreated nylon bed nets, and then respective homes received net treatments of diluted cow’s milk (placebo = 179) or 500 mg/m^2^ permethrin solution (treatment = 182). A four month post-treatment survey of each home, and each child, revealed nearly double the number of indoor crawling insects in homes provided placebo nets over those using the permethrin-treated bed nets, highlighting the broad impacts and ancillary benefits of net adoption [42]. Lines *et al*. [43] serendipitously reached similar conclusions when using permethrin-treated bed nets alongside treated curtains to suppress vector populations in Tanzania. Dead cockroaches, lice and bed bugs were seen throughout study huts and the researchers noted that ancillary pest management would likely appeal to communities more than simply controlling potential mosquito vectors.

Alongside the recorded success of Lines *et al*. [43] in Tanzania, Charlwood *et al*. [44] witnessed similar ancillary benefits. Following the distribution of permethrin impregnated nets for malaria vector control in Papua New Guinea, residents reported decreased bed bug and lice populations. Just 2 years later, and based on these reports, Charlwood & Dagoro [45] assessed the ability of IVC tools to suppress a major domiciliary pest, the bed bug, by distributing ITNs among workers in Papua New Guinea residing in a bed bug infested bunk house. They found that bed bug populations were indeed heavily suppressed for up to 12 weeks after net distribution, but began to rebound after residents using nets relocated, taking their nets with them. The researchers concluded that not only were the treated nets associated with decreases in bed bug populations, but that this ancillary benefit was the *main reason* why individuals wanted their nets reimpregnated with insecticide. This work, although small in scope, demonstrated the dual utility of IVC for the control of domiciliary pests.

Follow-up evaluations through the late 1900s and early 2000s demonstrated similar impacts of net adoption on pest populations. In a study of deltamethrin-treated curtains, 204 apartment residents were tasked with covering their windows for 1 h at both dawn and dusk and follow-up pest surveys were performed every two weeks for over a year. This intervention resulted in 88−94% reductions in surveyed mosquito vector species and 68−86% reduction in surveyed pest insect species [46]. Similarly, a study aimed to reduce plague incidence in Uganda, evaluated the efficacy of IRS against mosquitoes for controlling flea populations. Five villages were assigned to each of three groups: normal IRS (wall and ceiling application), modified IRS (wall, ceiling and a 1 m perimeter floor application) and a no-treatment control. They found that standard IRS application alone resulted in nearly 100% control of flea populations on surveyed rats [47]. These large impacts on domiciliary pests do not go unnoticed by communities, and researchers in Tanzania attempting to quantify community wide ITN impact saw this firsthand. Community members, each provided random nets treated with either permethrin or lambda-cyhalothrin (both pyrethroid insecticides) and surveyed to assess community acceptance and net impact, quickly began approaching members of the study team, ‘enthusiastically, and without prompting’, to inform them of declines in bed bug, flea and cockroach populations following net deployment. The control of these chronic infestations garnered support for the programme within the community, resulted in widespread net reimpregnation and led the researchers to conclude that the *main net benefits* were reductions in mosquito and bed bug populations [48].

These few studies highlight the impressive ability of IVC tools to secondarily suppress nuisance pests, but it is important to note that none of these studies demonstrated indoor pest elimination. This failure to eliminate pest populations, if left unaddressed, could potentially expose the same populations to strong recurrent selection pressure from AIs (figure 1). Nevertheless, the control of domiciliary pest insects is of high value to communities and represents a major ancillary benefit of IVC program adoption and adherence [49].

## 3. An intertwined history: shifting community perceptions underly indoor vector control longevity

Perceptions of ancillary benefits have been shown to shift the motivators for IVC programme participation within communities. Indeed, early reports on the perceptions and social factors associated with IVC adoption determined that many communities do so primarily as a reliable method of domiciliary pest control and only secondarily as a method of disease prevention and vector reduction [50–52]. Temu *et al*. [28] showed a clear example of this in Tanzania, where surveys were performed to assess relationships between bed bug infestations and IVC tool adoption. All surveyed residents from bed bug-infested homes, and over one-third of those from homes without bed bug infestations, found bed bugs to be *more* problematic than mosquitoes. The researchers showed that individuals from bed bug infested homes were not only significantly more likely to purchase and use pyrethroid-treated nets, but also had a 95% reimpregnation rate compared to 77% in uninfested homes [28]. They concluded that successful control of bed bugs (and other domiciliary pests) resulted in higher programme adherence, and that these benefits from the use of LLINs should be included in IVC information packages to gain community support.

Years of these collateral benefits have influenced the views of communities and persist today. A recent survey to assess awareness of insect vectors sampled >1000 households of varying socioeconomic backgrounds and education levels from all 10 districts in Botswana. They found that 58.9% of respondents were most concerned with indoor mosquitoes, but nearly 40% were most concerned with cockroaches and house flies [53]. It is easy to see how these two major and differing perceptions could arise in the same communities, and they hold the power to facilitate or stall IVC programme rollout and adoption. This is not surprising, as the adoption and success of any vector control programme is influenced not only by biological, climatic and socioeconomic factors but also by human perceptions [63]. The potential short transition from programmatic support to rejection in the wake of pest resurgence is not uncommon. While perceptions of IVC success are clearly rooted in lower vector and disease prevalence, often overlooked is the unintended suppression of non-target pests that can drive residents to enthusiastically adopt and maintain IRS and LLIN regimes (figure 1). Failing to recognize and value the sway of domiciliary pest control on community perceptions can impede IVC programmes even after measurable success is realized in their early adoption.

## 4. Indoor vector control programmes can select for insecticide resistance in domiciliary pests

The independent evolution of insecticide resistance in various populations of disease vectors is a major barrier to vector control programmes [64]. Likewise, heavy selection pressure through the overuse or misuse of insecticides favours insecticide resistance alleles in indoor pest populations. Two of the starkest examples of this are in the German cockroach (*Blattella germanica*) and the bed bug (*Cimex lectularius* and *Cimex hemipterus*).

The German cockroach, arguably the most impactful indoor pest on public health, burdens resource-constrained communities globally and its distribution is tied closely to the movements and behaviours of humans [65]. The introduction of German cockroaches is often accidental, or a result of spill-over in multi-unit or adjoining homes, and infestations can quickly reach thousands in the absence of effective control measures. Severe infestations result in large deposits of frass (shed exoskeletons, pulverized dead cockroaches and faecal matter), that contain high levels of allergens [66,67]. Prolonged exposure to these allergens has been shown to impact pulmonary health by contributing to the development of asthma [66,68]. Additionally, German cockroaches have been shown to harbour a diverse gut and faecal microbiome, implicating them in the transmission of pathogens under certain circumstances [67,69]. These burdens are exacerbated in low-income and resource-limited communities, especially when control measures are confounded by insecticide-resistant cockroach populations, despite the ability to mitigate these burdens with targeted, low-cost and proven control measures [68,70].

The bed bug, a persistent domiciliary pest that has resurged globally to unprecedented numbers in the past two decades, also places an immense burden on individuals and communities on nearly every continent [71–74]. Small founding propagules can quickly erupt into major infestations in the absence of effective eradication measures [75]. Although bed bugs are largely considered a nuisance pest, the bites of this obligatorily haematophagous insect are associated with allergic responses ranging from mild itching and swelling to inflamed and painful rashes, to pus-filled bullous lesions, and in rare cases, anaemia [76]. Further, bed bug faeces contain high levels of histamine, an immune modulator associated with itching, swelling and even anaphylaxis [77]. Because of these varying but potentially severe repercussions associated with bed bug infestations, their elimination from homes is a global priority.

The German cockroach and bed bug are highly adapted to human-built structures. They do not fly, have high-fidelity for recognized aggregation sites, and are rarely found in the outdoor environment (e.g. bed bugs associated with bats) (figure 1). As such, populations are isolated by the same artificial barriers that delimit our homes, with minimal gene flow between populations. Thus, adaptive alleles that confer resistance to insecticides quickly become fixed in these populations. In the case of the German cockroach, varying levels of resistance to 42 AIs have been shown dating back to the 1950s [78–81]. Similarly, varying levels of resistance to multiple classes of insecticides have been found in bed bug populations, including organochlorines, organophosphates, carbamates, pyrethroids, neonicotinoids and pyrroles [82]. Resistance has even been recorded to the phenylpyrazole class of insecticides in bed bugs, namely to the AI fipronil, despite no products containing fipronil being labelled for the control of bed bugs [83]. These examples highlight the remarkable propensity of these two pervasive indoor pests to rapidly evolve adaptive resistance mechanisms following the introduction of novel chemistries, and to maintain that resistance long after those insecticides are phased out of use.

The impact of IVC on domiciliary pests is not limited to German cockroaches and bed bugs, and it was widely documented across a variety of indoor pests in the 1960s by Busvine & Pal [84]. A WHO-designed survey was sent to more than 100 health authorities worldwide, and more than half responded. The surveys collectively indicated widespread insecticide resistance in many non-target species from regions with ongoing IVC, including in house flies and fleas. House fly populations across the world have evolved resistance to most products used in their control [85]. More relevant to VBDs, high resistance to a variety of insecticides has been observed in many flea populations, including the cat flea (*Ctenocephalides felis*), human flea (*Pulex irritans*) and likely as a result of DDT-based malaria efforts in the rat flea (*Xenopsylla cheopis*) (>5000-fold resistance) [86,87]. Flies and fleas pose direct risks to human health, often as co-infestations with other pests (figure 1), and such widespread resistance is at least in part a result of failing to consider the impact of IVC on indoor pests.

The WHO has documented the impact of IVC tools on domiciliary pest populations, and the resulting effect of indoor pests on vector control programmes. Indeed, a 1968 WHO publication [59] recognized that insecticide resistance typically emerges in indoor pest populations several years following application of IVC products. Therein, the WHO underscored that resistance in bed bug populations can lead to community interference with IVC application, as up to 80% of participants refused IRS reapplication. The solutions proposed included surveys, education programmes and insecticide treatments separate from malaria programmes. While each of the proposed strategies may have merit as part of an integrated bed bug control programme, it was not well integrated with IVC efforts [59]. In addition, the WHO evaluation and recommendations revolved around a single pest, despite the potential of other indoor pests to disrupt IVC. Recent WHO publications concerned with insecticide resistance and best use practices for IVC tools are nearly exclusively focused on the mitigation of insecticide-resistant vector populations [60–62], which is a critical component of IVC success. We posit, however, that decades of negligence in managing insecticide-resistant indoor pest populations in malaria endemic areas, and the limited current body of research on the continued impacts of IVC on pest populations, will equally hinder future IVC product development, rollout and success.

## 5. A dearth of contemporary research

We have summarized the interconnected history of IVC and domiciliary pests, the community expectation of IVC tools to control indoor pests and the propensity of obligate and opportunistic domiciliary pests to rapidly evolve insecticide resistance mechanisms when exposed to IVC measures. Yet, few contemporary researchers have investigated how well modern IVC tools control indoor pests. The lack of updated information jeopardizes the efficacy of IVC programmes, as many of these tools are used in conjunction with one another, overlapping with areas in the home frequented by non-target pests (figure 1).

Bed bugs, a principal example of this overlap, are the major focus of the minimal modern research that exists on IVC and indoor pest ecology. Owing to their need for a blood meal every few days, bed bugs rest in relative proximity to humans throughout their lives. A study performed in Magugu Tanzania assessed pyrethroid resistance in bed bugs (*C. lectularius*) through exposure to net swatches and pyrethroid treated filter papers. Tested AIs included varying concentrations of deltamethrin, permethrin, alpha-cypermethrin and lambda-cyhalothrin [54]. Of 1500 adult bed bugs assayed, ~50% were from homes without LLINs, but unfortunately the results were not stratified by LLIN use. The 24 h LD_95_ was between 3.3X (permethrin) and 15X (alpha-cypermethrin) higher than the estimated LD_50_. A reference-susceptible strain was not included, so resistance ratios could not be estimated, but extrapolating from other reports [88], these populations might have been highly resistant to permethrin. Similarly, the most recent LLIN bioefficacy study, performed in Cape Coast, Ghana, explicitly assessed LLIN efficacy for bed bug control. In a survey of 205 homes, 66.3% were found to be infested with bed bugs (*C. hemipterus*). Collected bed bugs were exposed to deltamethrin- or alpha-cypermethrin-impregnated LLINs for 72 h, and unsurprisingly only 4 and 12% mortality was seen, respectively [55]. Additional bed bugs from homes with low-level treatment were also assessed, but only 44–86% mortality was obtained. These studies present a case for pyrethroid resistance in bed bugs resulting in widespread failure of IVC tactics (including IRS and LLINs) to suppress bed bug populations.

Studies from other regions have demonstrated similar concerning trends following the use of LLINs. For example, Myamba *et al*. [56] investigated the resurgence of populations of *C. hemipterus* in villages in the Tanga region of Tanzania which had ongoing LLIN use for 6 years, with annual retreatment. Bed bugs collected from homes in five villages that adopted LLINs and five without LLINs, and their resistance to pyrethroid-treated bed nets and filter papers was assessed using contact assays. Shockingly, they saw only 4−51% mortality in bed bugs from villages with prolonged LLIN use, up to 72 h after a continuous 1 h exposure. In contrast, all but a single group of the bugs collected from control villages experienced >92% mortality. Similarly, researchers in India found 100% mortality in vector species after 3 min of LLIN exposure, but >30 min was needed for 100% mortality of house flies and American cockroaches (*Periplaneta americana*), and only 25% of bed bugs collected from local homes died after 30 min of exposure [57]. A critical consideration not addressed in these reports and rarely mentioned in related studies is how long indoor pests remain in contact with LLINs or IRS in residential settings (figure 1). The requirement of 30–60 min of continuous exposure to obtain 100% mortality is potentially unrealistic and fails to consider the complexities of pest behaviour and interactions with IVC tools.

Only one recent study addressed the behavioural interactions of an indoor pest with LLINs. It assessed the ability of various bed bug (*C. lectularius*) life stages, fed and unfed, of both insecticide susceptible and resistant strains to pass through several LLINs in pursuit of a blood meal or aggregation [58]. Most bed bugs regardless of life stage or feeding status were unhindered in their passage through two LLINs with differing weave and AIs, Olyset (permethrin) and PermaNet 2.0 (deltamethrin). Additionally, <30% mortality was found after 1 h potential exposure time in an insecticide susceptible strain during feeding assays and <65% mortality after 24−168 h potential exposure in aggregation assays. None of the pyrethroid-resistant bed bugs died in any of these assays. While it remains unclear whether bed bugs in the field engage in brief passages through the bed net in pursuit of blood meals and harbourage, or rest on the LLIN for extended periods of time, these results suggest the former. Notably, the biology of bed bugs dictates recurrent interactions with LLINs, which likely have been occurring for decades in malaria-endemic regions, and have exerted intense selection pressure on populations, driving the emergence of insecticide resistance and leading to anecdotal observations of bed bugs aggregating directly on LLINs.

Interactions of other haematophagous pests, such as fleas (*Ctenocephalides* and *Xenopsylla* spp.), with LLINs likely follow similar dynamics to bed bugs. However, in these holometabolous insects only adults are exposed to the LLIN selection pressures. Other household pests, such as the German cockroach and filth flies, are not expected to have frequent interactions with LLINs, and are likely affected more by IRS (figure 1). Nevertheless, the behavioural interactions of indoor pests with various IVC tools needs to be characterized, the repellency of IVC products and their AIs to indoor pests and resistant strains needs to be evaluated, and the distribution and frequency of resistant genotypes in indoor pest populations across malaria control programme sites needs to be quantified.

## 6. Intensifying selection pressures: adding fuel to the fire

Clear gaps in our understanding of the interactions of domiciliary pests with currently recommended IVC tools remain, yet the next generation of products are poised to dramatically intensify selection pressures, especially on bed bugs. First, novel LLIN designs incorporate synergists (inhibitors of detoxification enzymes, such as PBO) and multiple AIs to overcome insecticide-resistant vector populations. Indeed, the addition of PBO to LLINs increased mortality in highly pyrethroid-resistant mosquitoes in Uganda; however, bioefficacy of the bed nets declined quickly over the course of 2 years owing to dissipation of the synergist [89]. Despite this reported success, the addition of PBO to LLINs is not expected to provide substantial benefits against highly resistant mosquitoes. Similarly, varying success of the addition of PBO has been shown against highly resistant bed bug strains [62,83,90], which for decades have continued to develop extremely high levels of resistance to pyrethroids [55,91].

Another developing strategy to combat resistant vector populations is to incorporate new chemistry into both LLINs and IRS. For example, the combined use in Benin of alpha-cypermethrin (pyrethroid) treated LLINs (DuraNet) with each of three non-pyrethroid IRS (bendiocarb [carbamate], chlorfenapyr [pyrrole] and fipronil [phenylpyrazole]) significantly increased mortality in pyrethroid-resistant mosquitoes [92]. Varying levels of resistance to some of these potential products, including to the aforementioned insecticides, has already been seen in domiciliary pest populations [64,70,86,93–98]. Once again, the design and assessment of novel vector control strategies fails to consider the status of domiciliary pest populations, and how these products may confound control of these populations by further contributing to insecticide resistance. Therefore, we predict that indoor pest populations, namely bed bugs, will adapt to new LLIN and IRS faster than mosquito vectors owing to already high resistance, persistent interactions with IVC at all life stages and low gene flow between ‘closed’ sedentary populations. This rapid adaptation, together with insecticide-resistant vectors, increase the risks of net abandonment, individuals preferentially resting outdoors to escape homes inundated with biting pests, and a growing distrust of IVC products, including LLINs and IRS [99].

## 7. Concluding remarks: a pressing need

We have discussed the critical roles of indoor pest control and community perception for the early success and continued acceptance of IVC programmes and tools. In recent years, however, the fight against malaria has stalled and there is evidence of increasing malaria incidence in many communities [26,100]. The contribution of the unabated surge of insecticide-resistant domiciliary pests (figure 1) to the distrust and abandonment of IVC tools is unknown. The failure of emerging IVC technologies to consider their impacts on these pests dates back to the introduction of DDT and persists owing to limited AI formulations. This dearth of AIs with differing modes of action, and the long-term deployment of LLINs resulting in the delayed rollout of new products, place severe constraints on resistance management in the fight against malaria. There are no immediate solutions to this challenge. However, as a first step. we urge greater consideration of several key principles in the design, testing and implementation of current and proposed IVC tools:

— IVC products should be rigorously evaluated for their ability to simultaneously suppress vector and domiciliary pest populations.
— Greater emphasis should be placed on the discovery and development of inorganic AIs with physical or mechanical modes of action, such as silica and boric acid, which effectively control even highly insecticide-resistant bed bugs [101,102].
— The frequencies, levels and distribution of insecticide resistance in major indoor pests should be monitored in concert with monitoring of vector populations.
— The prevalence and burden of nuisance pests (filth flies), biting insects (bed bugs, fleas, lice) and obligatory indoor pests (bed bugs, German cockroach) should be assessed as part of IVC rollout and re-evaluation (figure 1).
— The changing perspectives and concerns of malaria endemic communities regarding IVC effectiveness should be repeatedly surveyed to ensure lasting adherence to new and established IVC technologies.

While this is not an exhaustive list, nor one that guarantees the success of IVC moving forward, it represents several crucial steps in our collective efforts to revitalize the stalled progress in the fight against malaria. IVC must take a more integrated approach, focusing not only on vector control but simultaneously on indoor health and wellbeing, including indoor pest management, an approach that will more effectively support the goal of malaria elimination.

## Data Availability

This article has no additional data.
